# Factors affecting the medication literacy of older adults and targeted initiatives for improvement: a cross-sectional study in central China

**DOI:** 10.3389/fpubh.2023.1249022

**Published:** 2024-01-16

**Authors:** Chao Mei, Baoli Xu, Xuefeng Cai, Min Wan, Zhigang Zhao, Yongning Lv, Yu Zhang, Ruxu You

**Affiliations:** ^1^Department of Pharmacy, Union Hospital, Tongji Medical College, Huazhong University of Science and Technology, Wuhan, China; ^2^Department of Pharmacy, People’s Hospital of Dongxihu District, Wuhan, China; ^3^Department of Pharmacy, Beijing Tiantan Hospital, Capital Medical University, Beijing, China

**Keywords:** medication literacy, older adults, drug therapy, questionnaire survey, aging

## Abstract

**Introduction:**

This study explored the state of rational drug use among older adults in central China, aiming to unveil factors influencing their medication literacy and proposing targeted improvement measures.

**Methods:**

A cross-sectional study involving 454 participants aged 60 and above was conducted in Hubei province between February 1 and May 30, 2023, with data collected through face-to-face interviews by pharmacists. Multiple logistic regression analysis was conducted to determine factors that affected medication literacy.

**Results:**

Of the 412 valid questionnaires, findings revealed inadequate knowledge of rational drug use among older adults in central China. Those who fully understood (105, 25.49%, OR = 9.349, *p* < 0.001, 95%CI = 3.884–22.502) or partially understood (228, 55.34%, OR = 3.295, *p* = 0.002, 95%CI = 1.548–7.013) drug instructions exhibited significantly higher medication literacy than those who did not understand (79, 19.17%). Subsequent research revealed a lack of awareness in reading drug instructions or difficulty in understanding them. Most older adults seldom heard of but exhibited high acceptance of medication guidance services.

**Discussion:**

In conclusion, the ability to comprehend drug instructions significantly influenced the medication literacy of older adults. Initiatives such as revising age-appropriate drug instructions could effectively enhance rational drug use among this demographic.

## 1 Introduction

Irrational use of medicines, manifested as drug abuse, insufficient or excessive drug dosage, repeated drug use and inappropriate combination of drugs, poses a pervasive global health challenge (1, 2). Alarmingly, more than half of the drugs are prescribed, dispensed or sold improperly, and only half of patients take drugs in a rational way (3–6). This irrational medication use can lead to adverse reactions, drug resistance, addiction, complications, drug interactions, prolonged illnesses, and adverse economic consequences, significantly endangering public health (6, 7). Consequently, enhancing public medication literacy and disseminating knowledge on rational drug use have become pivotal aspects of healthcare initiatives.

The United Nations predicted that by 2050, one in five individuals will be aged 65 or older, highlighting the escalating global concern of aging (8, 9). Aging is defined as a progressive decline of physical function over time and ultimately increased susceptibility to death (10). Older adults possess the characteristics of reduced cognitive ability, physiological function, immunity, and metabolic rate, etc. (11, 12). Up to 75.8% of the residents aged ≥60 years had at least one chronic disease in China (13). Given their propensity for suffering from multiple diseases and concurrently taking various drugs, older adults represent a main population of irrational drug use (14–16). Therefore, it is necessary to understand the current situation of rational drug use among older adults and to analyze factors affecting their medication safety (14, 17).

Medication literacy refers to the cognitive and ability to access, comprehend, and process medication-related information, which is crucial for using drugs correctly in terms of timing and dosage (18, 19). It is widely accepted that personal and contextual factors affect the way individuals obtain and maintain medication literacy skills (20). However, limited studies have explored the essential factors contributing to medication literacy of older adults in China (21–24).

By performing a cross-sectional survey, this study aimed to investigate the drug usage patterns of older adults in central China and identify factors that affected their medication literacy. The study was conducted by professional pharmacists in the form of face-to-face interviews. The survey lasted more than 30 min for each participant, which greatly improved the accuracy of the survey results. The findings of this study provide valuable insights into the current state of rational drug use of Chinese older adult population, paving the way for targeted interventions to improve medication literacy and promote rational drug use in this demographic.

## 2 Methods

### 2.1 Study design

This study research is an integral component of a national project in China (project name: Foreign drug aging system research and reference significance for China; No.: 2023-018), held by the China Society for Drug Regulation (CSDR). The cross-sectional study conducted in Hubei province, a provincial-level administrative region located in central China, was analyzed in this study. The survey was conducted between February 1 and May 30, 2023, and the method of regional stratified sampling was used in this study. The sample size, determined through power analysis for 90 percent power, was established at 400 subjects. A total of 454 older adults in Hubei province participated in the questionnaire survey. The informed consent had been obtained from all the participants before the study was initiated. The survey was administered through face-to-face interviews, each lasting over 30 min. During the interview, pharmacists communicated with patients according to the electronic questionnaire, and all contents of the questionnaire were filled in by pharmacists.

### 2.2 Inclusion criteria

The participant inclusion criteria were defined as follows: (1) age of 60 years or older; (2) residing in urban areas of Hubei Province, China; (3) suffering from chronic diseases (such as hypertension, diabetes, hyperlipidemia, hyperuricemia, gout, asthma, chronic obstructive pulmonary disease and cancer, etc.); (4) consenting to the home survey conducted by pharmacists; and (5) possessing effective communication skills and displaying active cooperation during the survey. A total of 454 residents completed the questionnaire. After excluding the incomplete questionnaires with missing value ≥10% and those who did not meet the inclusion criteria, 412 questionnaires were finally included in this study.

For the pharmacists involved, the inclusion criteria comprised: (1) holding a junior or higher professional title qualification; (2) possessing over 5 years of practical experience; and (3) having a background in providing community pharmaceutical services or engaging in drug consultation services.

### 2.3 Questionnaire content

The questionnaire was meticulously crafted by a special expert group comprising experts in pharmacy, management, social studies, and questionnaire management. The design process incorporated a thorough literature review, expert consultations, and group discussions. The survey consisted of two parts: an impact factor questionnaire and a medication literacy scale. The impact factor scale gathered information on participants’ demography characteristics, health status, disease types and indicators, drug use situations and other information. The medication literacy scale was used to quantify the ability of scientific drug use of older adults. This questionnaire consisted of 55 items, which covered the general knowledge of drug instruction usage, medication compliance, drug storage, disposal of expired drugs, unreasonable drug use behavior and management of adverse drug reactions, etc. Each participant’s medication literacy score was assessed based on the total points from these 55 questions, where a higher score indicated superior medication literacy. Detailed information and scores for each answer were consolidated in Supplementary Tables S1, S2. Questions 1–26 and 33–55 were scored on a five-point Likert scale, while questions 27–32 were true or false queries. The total points were calculated and divided into two categories for the purpose of logistic regression modeling. To establish the cutoff value, the focus group discussion method was employed. A special expert group was gathered together to express and discuss their attitudes, perceptions, opinions, and experiences on value setting, which synthetically considered the score situation of medication literacy of older adults in Hubei Province and the whole country. A total score of ≥183 was designated as meeting the standard for medication literacy, while scores below this threshold were considered substandard.

### 2.4 Data collection

The study was approved by the Ethics Committee of Beijing Tiantan Hospital, Capital Medical University (registration number: KY2022-228-01). Prior to the commencement of the study, explicit informed consent was obtained from all participants. The survey was conducted by professional pharmacists in the form of face-to-face interviews. A total of 454 older adults in Hubei province accepted the survey conducted by pharmacists. It took more than 30 min to fill out the data forms. The survey included an impact factor questionnaire and a medication literacy scale. Questionnaires with missing values equal to or exceeding 10% and those failing to meet the inclusion criteria were excluded from the analysis.

### 2.5 Statistical analysis

The sample size of the study was determined through a power analysis based on 90% power. Cronbach’s Alpha was employed to evaluate the confidence of the survey. The collected data were analyzed using the SPSS (version 25.0) statistical software package, including the general description analysis, chi-square test and multiple logistic regression analysis. The chi-square test was used to evaluate the relationship between medication literacy and each independent variable. The multiple logistic regression analysis was used to determine the factors that significantly affect medication literacy of the older adults. Participants’ medication literacy was set as the dependent variable. The education level, living alone or not, working status, monthly income, disease status, knowledge of disease-related indexes, understanding of drug instructions, as well as the history of adverse drug reaction were taken as independent variables. The gender and age of participants were set as covariates. The entry level was set at 0.05 and the exclusion level at 0.10. *p* < 0.05 was considered statistically significant.

## 3 Results

### 3.1 Participant characteristics

Cronbach’s Alpha was used to evaluate the confidence of the survey and the result was 0.931, indicating a high level of reliability. Following quality assessment, a total of 412 valid questionnaires were retained, achieving an acceptability ratio of 90.75%. The basic characteristics of participants were summarized in Table 1, the gender distribution was nearly equal, with 209 female participants (50.73%) and 203 male participants (49.27%). The average age of older adults was 68.69 years, and a significant majority (65.78%) were under the age of 70. 94.91% of participants had received at least a primary school education. Moreover, 91.99% of older adults lived with other relatives, and 70.39% were retired and stayed at home. The monthly income of most participants (258, 62.62%) was between 1,000 to 5,000 yuan. Additionally, 60.19% of participants had more than one type of chronic disease.

**Table 1:** Distribution of basic characteristics of participants.

Characteristics		*N*	%
Gender	Female	209	50.73%
	Male	203	49.27%
Age	60–70	271	65.78%
	71–80	88	21.36%
	81~	53	12.86%
Education level	Illiteracy	21	5.10%
	Primary/Junior high school	241	58.50%
	Senior high school and above	150	36.41%
Living alone	Yes	33	8.01%
	No	379	91.99%
Working status	Farmer or migrant worker	114	27.67%
	Retirement	290	70.39%
	Re-employed	8	1.94%
Monthly income	0–1,000	88	21.36%
	1,000–5,000	258	62.62%
	5,000~	66	16.02%
Number of chronic diseases	1	164	39.81%
	2–3	220	53.40%
	4~	28	6.80%

### 3.2 Assessment of older adults’ medication literacy

Participants’ medication literacy was determined based on the outcomes of the medication literacy scale (Table 2). Among the 412 subjects, 192 (46.60%) older adults demonstrated satisfactory medication literacy, while 220 (53.40%) of them exhibited insufficient knowledge regarding rational drug use. Further analysis revealed that medication literacy among males was significantly higher than that of females (*p* = 0.040), while age had no obvious impact on medication literacy of older adults (*p* = 0.226). In addition, medication literacy significantly increased with educational attainment (*p* = 0.000). Only 19.05% of illiterate older adults met the standards, while the rate of older adults with primary/junior high school and senior high school or above education level were 39.00% and 62.67%, respectively. Older adults living alone (33.33%) exhibited relatively lower medication literacy than those living with families (47.76%), but no significant statistical difference was observed (*p* = 0.111). Occupationally, 87.50% of re-employed older adults met the medication literacy standard, compared to 48.97% for retired older adults and 37.72% for farmers or migrant workers (*p* = 0.008). In addition, we also found that medication literacy was positively correlated with monthly income (*p* = 0.001). Among older adults with monthly income of less than 1,000, 1,000 to 5,000 and over 5,000 yuan, the proportions of medication literacy reaching the standard were 31.82, 47.67, and 62.12%, respectively. Regarding health, the number of chronic diseases had no impact on medication literacy (*p* = 0.646). Older adults who can fully understand the knowledge of disease-related indexes (58.02%) exhibited higher medication literacy than those who can partially (47.73%) or cannot understand (28.36%) (*p* = 0.001). It is worth noting that the ability to understand drug instructions had a significant impact on medication literacy of older adults (*p* = 0.000). Among older adults who fully understood drug instructions, 74.29% met the standard, while those who partially understood and could not understand achieved rates of 43.86% and 17.72%, respectively. Finally, older adults who had experienced adverse drug reactions (51.96%) demonstrated slightly lower medication literacy than those who had not (55.31%) (*p* = 0.056).

**Table 2:** Comparison of medication literacy of the older adult with different characteristics.

Characteristics		Total *N*	Medication literacy scale		χ2	*p*
			*N*. (183)	%		
Gender	Female	209	87	41.63%	4.219	0.040
	Male	203	105	51.72%		
Age	60–70	271	131	48.34%	2.974	0.226
	71–80	88	34	38.64%		
	81~	53	27	50.94%		
Education level	Illiteracy	21	4	19.05%	27.554	0.000
	Primary/Junior high school	241	94	39.00%		
	Senior high school and above	150	94	62.67%		
Living alone	Yes	33	11	33.33%	2.538	0.111
	No	379	181	47.76%		
Working status	Farmer or migrant worker	114	43	37.72%	9.643	0.008
	Retirement	290	142	48.97%		
	Re-employed	8	7	87.50%		
Monthly income	0–1,000	88	28	31.82%	14.236	0.001
	1,000–5,000	258	123	47.67%		
	5,000~	66	41	62.12%		
Number of chronic diseases	1	164	73	44.51%	0.874	0.646
	2–3	220	104	47.27%		
	4~	28	15	53.57%		
Knowledge of disease-related indexes	Unclear	67	19	28.36%	13.343	0.001
	Partially understand	264	126	47.73%		
	Fully understand	81	47	58.02%		
Ability of understanding drug instruction	Unable	79	14	17.72%	59.506	0.000
	Partially understand	228	100	43.86%		
	Able	105	78	74.29%		
History of adverse drug reaction	No	179	99	55.31%	3.645	0.056
	Yes	233	93	51.96%		

### 3.3 Multiple logistic regression analysis of factors affecting medication literacy

Based on the above analysis, multiple logistic regression analysis was conducted to discern the pivotal factors significantly influencing medication literacy of older adults. The detailed variable assignments for the multiple logistic regression analysis were outlined in (Supplementary Table S3). The Hosmer-Lemeshow test regression analysis results yielded a χ^2^ value of 9.198 and a *p*-value of 0.326, indicating that the regression model possessed a high fitting degree.

The results of multiple logistic regression analysis were presented in Table 3. According to the results, medication literacy of older adults aged over 81 was significantly higher than that of aged 60–70 (OR = 2.583, *p* = 0.013, 95%CI = 1.223–5.454), while no significant difference was observed in the 71–80 age group (OR = 0.910, *p* = 0.750, 95%CI = 0.512–1.620). The ability to comprehend drug instructions emerged as a significant factor influencing participants’ medication literacy. Those who fully understood (OR = 9.349, *p* < 0.001, 95%CI = 3.884–22.502) or partially understood drug instructions (OR = 3.295, *p* = 0.002, 95%CI = 1.548–7.013) exhibited markedly higher medication literacy compared to older adults who could not understand them. Additionally, older adults without a history of adverse drug reactions demonstrated superior medication literacy compared to those with a history (OR = 0.524, *p* = 0.006, 95%CI = 0.330–0.830). Other factors such as gender, education level, whether living alone, working status, monthly income, disease status and knowledge of disease-related indexes showed no significant impact on medication literacy of older adults.

**Table 3:** Multiple logistic regression analysis of factors affecting the older adults’ medication literacy.

Variables		*β*	S.E.	Wald	*p*	OR	95%CI
Gender		−0.246	0.241	1.041	0.308	0.782	0.488–1.254
Age	60–70			7.227	0.027		
	71–80	−0.094	0.294	0.102	0.750	0.910	0.512–1.620
	81~	0.949	0.381	6.195	0.013	2.583	1.223–5.454
Education level	Illiteracy			2.672	0.263		
	Primary/Junior high school	0.141	0.704	0.040	0.841	1.152	0.290–4.580
	Senior high school and above	0.577	0.754	0.586	0.444	1.781	0.406–7.801
Living alone		0.497	0.433	1.317	0.251	1.643	0.704–3.837
Working status	Farmer or migrant worker			1.506	0.471		
	Retirement	0.042	0.283	0.022	0.882	1.043	0.598–1.817
	Re-employed	1.391	1.135	1.501	0.221	4.019	0.434–37.208
Monthly income	0–1,000			0.412	0.814		
	1,000–5,000	0.189	0.312	0.365	0.546	1.208	0.655–2.227
	5,000~	0.241	0.440	0.301	0.583	1.273	0.537–3.015
Number of chronic diseases	1			1.622	0.444		
	2–3	0.231	0.239	0.937	0.333	1.260	0.789–2.012
	4~	0.514	0.472	1.186	0.276	1.673	0.663–4.221
Knowledge of disease-related indexes	Unclear			1.807	0.405		
	Partially understand	0.419	0.339	1.531	0.216	1.521	0.783–2.956
	Fully understand	0.511	0.418	1.494	0.222	1.667	0.735–3.781
Ability of understanding drug instruction	Unable			26.113	0.000		
	Partially understand	1.192	0.385	9.572	0.002	3.295	1.548–7.013
	Able	2.235	0.448	24.880	0.000	9.349	3.884–22.502
History of adverse drug reaction		−0.647	0.235	7.578	0.006	0.524	0.330–0.830

### 3.4 Existing problems and suggestions of the drug instruction

The results from multiple logistic regression analysis indicated that the most significant impact on medication literacy of older adults was the ability to understand drug instructions. Consequently, we delved deeper into whether older adults have enough consciousness to self-medicate according to drug instructions.

**Figure 1:**
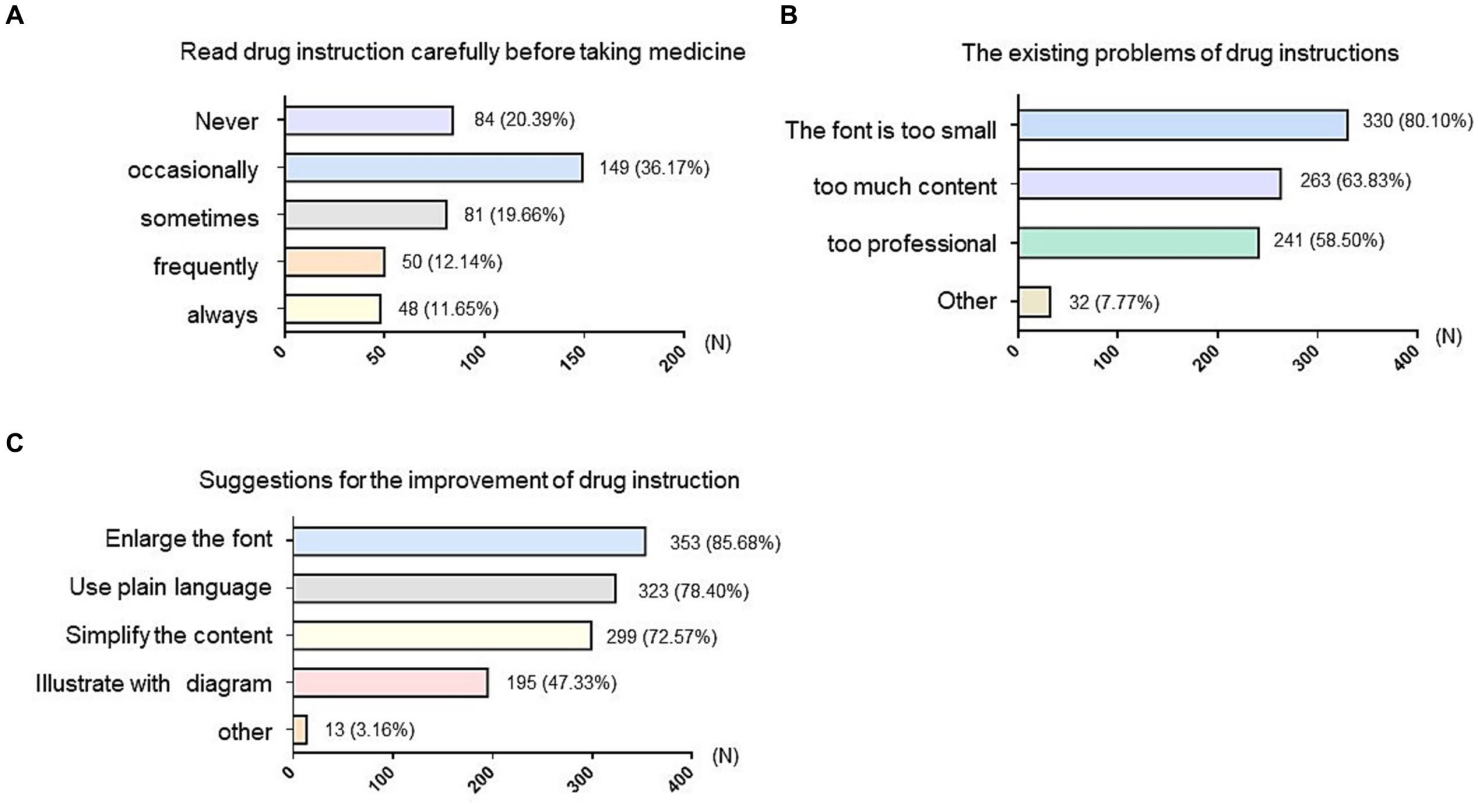
Investigation of existing problem and suggestions of the drug instruction among older adults. **(A)** Survey on whether older adults read the instruction carefully before taking medicine. **(B)** Survey of the existing problems that older adults thought the instruction has. **(C)** The suggestions for the improvement of the drug instruction provided by older adults. The vertical axis represented different options and the horizontal axis indicated the number (N) of people who chose that option. The numbers on the bar chart represent the number and percentage of people who chose the corresponding option.

Among the 412 subjects included in this study, only 48 (11.65%) of them always carefully read the drug instructions before taking drugs. 50 (12.14%) and 81 individuals (19.66%) often and sometimes read drug instructions, respectively. More than half of older adults only occasionally (149, 36.17%) or never (84, 20.39%) read drug instructions (Figure 1A). These findings underscored a prevalent lack of consciousness among older adults regarding rational medication adherence based on drug instructions. It is of great significance to enhance public awareness and education to promote the concept of safe medication practices in alignment with drug instructions.

Beyond the general reluctance to recognize the necessity of reading drug instructions, another obstacle lies in the difficulty of understanding the contents. According to our survey, the specific difficulties include: (1) a vast majority of older adults (330, 80.10%) found the font size in drug instructions too small to read clearly; (2) 263 (63.83%) individuals pointed out that the abundance of content in drug instructions made it challenging to identify crucial information; and (3) 241 respondents (58.50%) found the technical wording too complex to comprehend (Figure 1B). Consistently with the above issues, more than 70% participants recommended enlarging the font size (353, 85.68%); 299 (72.57%) people proposed simplifying drug instructions to highlight essential information; 323 (78.40%) people advocated for expressing content in plain language; 195 (47.33%) suggested incorporating diagrams to illustrate information (Figure 1C).

### 3.5 Investigation of the pharmaceutical service status among the older adults

**Figure 2:**
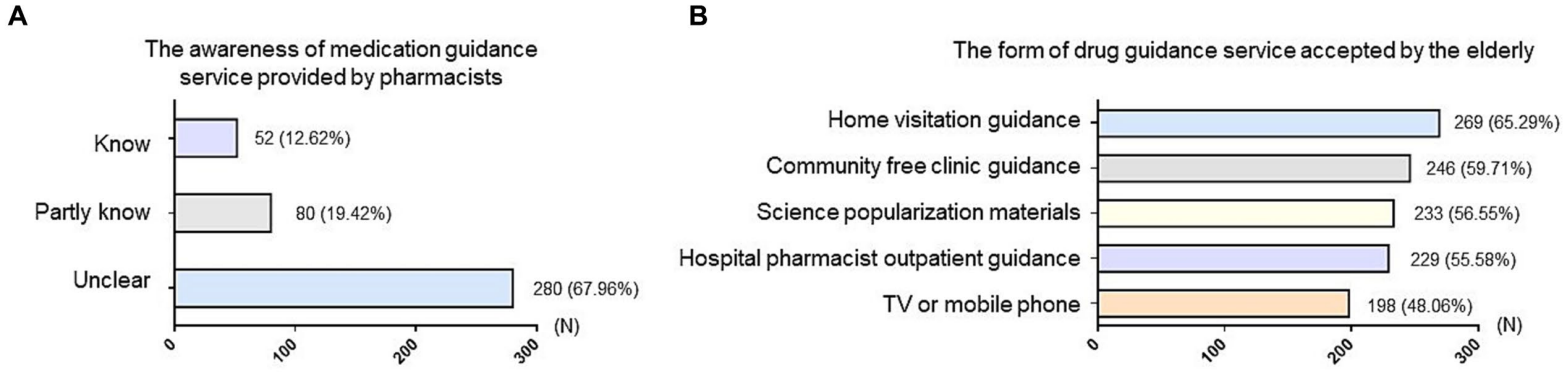
Investigation of the popularity of pharmaceutical service status among older adults. **(A)** Survey on the awareness of medication guidance service provided by pharmacists among older adults. **(B)** Survey of the form of drug guidance service accepted by older adults. The vertical axis represented different options and the horizontal axis indicated the number (N) of people who chose that option. The numbers on the bar chart represent the number and percentage of people who chose the corresponding option.

The knowledge and acceptance of medication guidance services provided by pharmacists were also investigated in the older adults. Unfortunately, most older adults (280, 67.96%) reported never having heard of the medication guidance services provided by pharmacists, underscoring the inadequacy of publicizing pharmaceutical services in central China (Figure 2A).

Actually, most older adults exhibited a high acceptance of various medication guidance services, such as household medication guidance provided by pharmacists (269, 65.29%), community free clinic guidance (246, 59.71%), introduction on science popularization and publicity materials (233, 56.55%), guidance of pharmaceutical clinic at hospital (229, 55.58%), and the introduction on TV or mobile phone (198, 48.06%) (Figure 2B). Leveraging these channels in future initiatives can effectively enhance the outreach and impact of medication guidance services, contributing to the safer and more rational use of medications among older adults.

## 4 Discussion

This study conducted a household survey to assess medication literacy of 412 older adults in central China, revealing a notable deficiency in their knowledge of rational drug use. Statistical analyses identified the ability to read drug instructions as the most influential factor impacting medication literacy of older adults. A strategic initiative to revise age-appropriate drug instructions emerged as a promising approach to enhance the rational use of medications in this demographic.

Given the aging population and heightened health awareness, the growing demand for medical services among older adults underscored the crucial need to evaluate their medication literacy and proposed effective improvement measures to prevent adverse drug reactions (25–29). Previous researches had highlighted the susceptibility of older adults to the risks of irrational medication due to their tendency to suffer from multiple diseases and take multiple medications concurrently (30). Coupled with their unique characteristics, older adults are highly susceptible to the harm of irrational medication (31–33). Compared with some developed countries, we still need more attention and research in improving medication literacy of older adults in China (18). Up to now, there are only a few studies focused on medication literacy and its influence factors in Chinese older adults, especially the quantitative research results of medication literacy (34–36). Limited studies suggested that medication literacyis related to individual factors and medical factors (35). Individual factors mainly include age, social support, education level and so on. Medical factors refer to inappropriate communication of medication problems between medical staffs and patients (34). Our study revealed a novel finding that the ability to understand drug instructions significantly influenced medication literacy of older adults, surpassing the impact of other individual and medical factors. This discovery held promise for implementing targeted modifications to effectively enhance medication literacy.

As drug instructions serve as the primary conduit for drug safety information, offering critical guidance for the safe and rational use of medications, our results prompted several recommendations for improvement: (1) Drug instructions could be divided into “professional” and “patient” versions. The patient version is designed specifically for patients and should be simplified to only include what patients must know, such as the drug name, ingredients, indication, contraindication, usage, dosage, storage, adverse reaction and manufacturer, etc.; (2) Improve the legibility and understandability of drug instructions by avoiding technical terms and using plain language; (3) Describe and display the contents of drug instructions vividly by exhibiting schematic diagrams; (4) Enlarge font size for improved readability; (5) Adjust and clearly label the usage and dosage according to the physiological characteristics of the older adults; and (6) Incorporate a QR code for easy access to video/audio explanations when scanned with a mobile phone.

In addition to drug instructions, we also observed that medication literacy of older adults increased with their education level, working status, monthly income and the knowledge of disease-related indexes. Moreover, males exhibited higher medication literacy than females. Future studies with an expanded sample size are recommended to ascertain the significant impact of these factors on medication literacy in older adults (24, 37, 38).

## Data availability statement

The original contributions presented in the study are included in the article/Supplementary material, further inquiries can be directed to the corresponding author.

## Ethics statement

The studies involving humans were approved by The Ethics Committee of Beijing Tiantan Hospital, Capital Medical University (registration number: KY2022-228-01). The studies were conducted in accordance with the local legislation and institutional requirements. The participants provided their written informed consent to participate in this study.

## Author contributions

RY, YL, ZZ, and YZ contributed to the design of the study. BX, MW, and XC provided help for data collection. CM performed data analysis and manuscript write up. All authors contributed to the article and approved the submitted version.
